# Supplementary material for: Deep learning empowered volume delineation of whole-body organs-at-risk for accelerated radiotherapy
Source: Nat Commun. 2022 Nov 2;13:6566. doi: 10.1038/s41467-022-34257-x (PMC9630370; doi:10.1038/s41467-022-34257-x)
Supplement: Supplementary file 1 — Supplementary Information [file 41467_2022_34257_MOESM1_ESM.pdf]

Supplementary Information for

Deep learning empowered volume delineation of whole-body organs-at-risk for accelerated radiotherapy

Feng Shi[1,#], Weigang Hu[2,3,#], Jiaojiao Wu[1,#], Miaofei Han[1], Jiazhou Wang[2,3], Wei Zhang[4], Qing Zhou[1], Jingjie Zhou[4], Ying Wei[1], Ying Shao[1], Yanbo Chen[1], Yue Yu[1], Xiaohuan Cao[1], Yiqiang Zhan[1], Xiang Sean Zhou[1], Yaozong Gao[1,*], Dinggang Shen[5,1,6,*]

[1]*Department of Research and Development, Shanghai United Imaging Intelligence Co., Ltd., Shanghai, China*

[2]*Department of Radiation Oncology, Fudan University Shanghai Cancer Center, Shanghai, China*

[3]*Department of Oncology, Shanghai Medical College, Fudan University, Shanghai, China*

[4]*Radiotherapy Business Unit, Shanghai United Imaging Healthcare Co., Ltd., Shanghai, China*

[5]*School of Biomedical Engineering, ShanghaiTech University, Shanghai, China*

[6]*Shanghai Clinical Research and Trial Center, Shanghai, China*

[#] F. Shi, W. Hu, and J. Wu contributed equally to this work.

[*] Corresponding authors: Yaozong Gao, yaozong.gao@uii-ai.com

Dinggang Shen, Dingang.Shen@gmail.com

The PDF file includes:

Supplementary Figs. 1 to 3

Supplementary Tables 1 to 6

Supplementary References

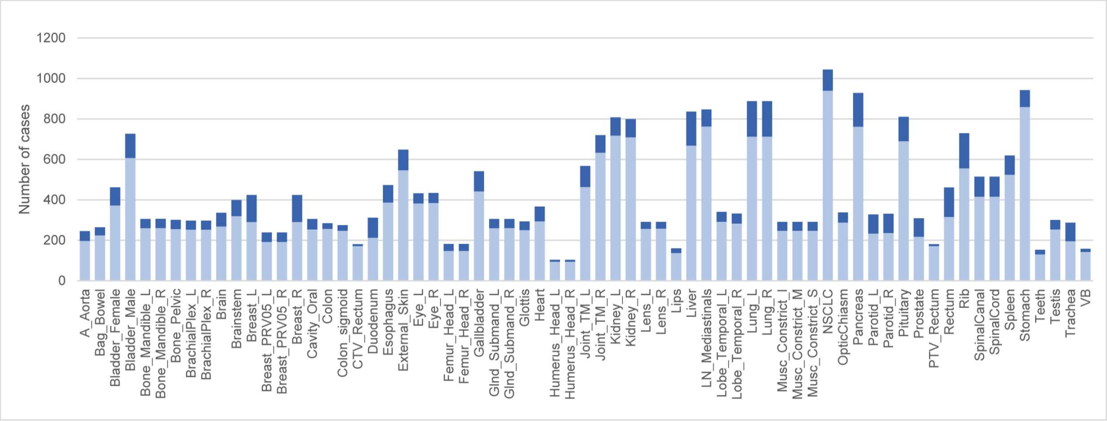

Supplementary Fig. 1. RTP-Net is evaluated on a large-scale dataset of 28,581 cases for 67 segmentation tasks, from which 28,219 cases are OAR-related and the rest are tumor-related. In each bar, the darker one represents the testing set, and the lighter one represents the training set.

Supplementary Table 1. The Dice coefficients of RTP-Net in segmenting whole-body OARs. Each Dice coefficient is represented with a mean and 95% confidence interval (CI).

No.	Head part	Dice coefficient	No.	Chest part	Dice coefficient
1	Brain	0.993 (0.992, 0.994)	1	Lung_L	0.988 (0.988, 0.989)
2	Lens_L	0.985 (0.975, 0.995)	2	Lung_R	0.988 (0.988, 0.989)
3	Eye_R	0.977 (0.974, 0.981)	3	Esophagus	0.975 (0.962, 0.988)
4	Eye_L	0.972 (0.966, 0.977)	4	Humerus_Head_L	0.972 (0.961, 0.983)
5	Bone_Mandible_R	0.952 (0.946, 0.958)	5	Humerus_Head_R	0.971 (0.960, 0.982)
6	Bone_Mandible_L	0.951 (0.944, 0.959)	6	Heart	0.969 (0.962, 0.976)
7	Parotid_R	0.951 (0.947, 0.956)	7	VB	0.969 (0.964, 0.975)
8	Brainstem	0.941 (0.938, 0.945)	8	Breast_R	0.968 (0.964, 0.971)
9	Cavity_Oral	0.924 (0.916, 0.932)	9	Trachea	0.960 (0.954, 0.965)
10	Parotid_L	0.905 (0.897, 0.914)	10	Breast_PRV05_L	0.947 (0.939, 0.954)
11	Lens_R	0.892 (0.871, 0.912)	11	Breast_PRV05_R	0.942 (0.935, 0.950)
12	Joint_TM_L	0.886 (0.866, 0.906)	12	Breast_L	0.937 (0.933, 0.941)
13	Joint_TM_R	0.856 (0.838, 0.874)	13	A_Aorta	0.934 (0.914, 0.954)
14	Glnd_Submand_R	0.852 (0.833, 0.872)	14	Rib	0.933 (0.930, 0.935)
15	Teeth	0.845 (0.816, 0.873)	15	NSCLC	0.858 (0.834, 0.883)

16	Lips	0.844 (0.827, 0.861)
17	Lobe_Temporal_L	0.843 (0.830, 0.857)
18	Lobe_Temporal_R	0.840 (0.826, 0.853)
19	Glnd_Submand_L	0.833 (0.805, 0.861)
20	Musc_Constrict_I	0.804 (0.788, 0.821)
21	Glottis	0.798 (0.782, 0.815)
22	Musc_Constrict_S	0.784 (0.768, 0.800)
23	Musc_Constrict_M	0.737 (0.710, 0.763)
24	Pituitary	0.736 (0.717, 0.754)
25	OpticChiasm	0.632 (0.602, 0.662)
26	BrachialPlex_R	0.607 (0.586, 0.629)
27	BrachialPlex_L	0.603 (0.578, 0.628)

No.	Pelvic cavity part	Dice coefficient
1	Bone_Pelvic	0.982 (0.978, 0.987)
2	Femur_Head_R	0.981 (0.978, 0.985)
3	Femur_Head_L	0.973 (0.970, 0.975)
4	Bladder_Male	0.955 (0.944, 0.966)
5	Rectum	0.937 (0.928, 0.946)
6	Testis	0.913 (0.890, 0.937)
7	Bladder_Female	0.902 (0.874, 0.931)
8	Prostate	0.899 (0.888, 0.909)
9	Colon_sigmoid	0.846 (0.805, 0.886)

16	LN_Mediastinals	0.606 (0.571, 0.640)

No.	Abdomen part	Dice coefficient
1	Liver	0.980 (0.977, 0.983)
2	Kidney_L	0.979 (0.972, 0.985)
3	Kidney_R	0.978 (0.975, 0.980)
4	Stomach	0.978 (0.971, 0.985)
5	Bag_Bowel	0.973 (0.970, 0.976)
6	Spleen	0.969 (0.965, 0.973)
7	Gallbladder	0.944 (0.936, 0.953)
8	Pancreas	0.907 (0.898, 0.916)
9	Colon	0.874 (0.839, 0.910)
10	Duodenum	0.837 (0.818, 0.857)

No.	Whole body	Dice coefficient
1	SpinalCanal	0.939 (0.934, 0.944)
2	SpinalCord	0.911 (0.897, 0.924)
3	External_Skin	0.997 (0.997, 0.997)

Supplementary Table 2. Dice coefficients of eight segmentation tasks by our proposed RTP-Net, U-Net, nnU-Net, and Swin UNETR. The dice coefficient is represented with mean and 95% CI. Statistical analyses are performed using two-way ANOVA followed by Dunnett's multiple comparison tests. The number of eight organs can be referred to Supplementary Fig. 1. The two-tailed adjusted p values of Dice coefficients between RTP-Net and other three methods (U-Net, nnU-Net, and Swin UNETR) are 0.596, 0.999, and 0.965 for brain segmentation, respectively; < 0.001, 0.234, and 0.001 for brainstem segmentation, respectively; 0.206, 0.181, and 0.183 for rib segmentation, respectively; 0.367, 0.986, and 0.010 for heart segmentation, respectively; 0.002, 0.999, 0.003 for liver segmentation, respectively; 0.991, 0.900, and 0.803 for pelvic segmentation, respectively; < 0.001, 0.010, and 0.003 for rectum segmentation, respectively; 0.999, 0.827, and 0.932 for bladder segmentation, respectively.

	RTP-Net	U-Net	nnU-Net	Swin UNETR	$P_{(RTP\text{-}Net\ vs.\ U\text{-}Net)}$	$P_{(RTP\text{-}Net\ vs.\ nnU\text{-}Net)}$	$P_{(RTP\text{-}Net\ vs.\ Swin\ UNETR)}$
Brain	0.993 (0.992, 0.994)	0.901 (0.847, 0.956)	0.994 (0.993, 0.995)	0.976 (0.946, 1.000)	0.596	0.999	0.965
Brainstem	0.941 (0.938, 0.945)	0.915 (0.903, 0.926)	0.930 (0.926, 0.934)	0.916 (0.912, 0.921)	< 0.001	0.234	0.001
Rib	0.939 (0.936, 0.941)	0.925 (0.921, 0.928)	0.941 (0.938, 0.945)	0.924 (0.921, 0.928)	0.206	0.181	0.183
Heart	0.969 (0.962, 0.976)	0.928 (0.893, 0.963)	0.967 (0.962, 0.971)	0.947 (0.937, 0.958)	0.367	0.986	0.010
Liver	0.980 (0.977, 0.983)	0.963 (0.953, 0.973)	0.980 (0.976, 0.983)	0.964 (0.959, 0.969)	0.002	0.999	0.003
Pelvis	0.982 (0.978, 0.987)	0.980 (0.976, 0.984)	0.977 (0.955, 0.987)	0.976 (0.972, 0.979)	0.991	0.900	0.803
Rectum	0.937 (0.928, 0.946)	0.824 (0.795, 0.853)	0.921 (0.913, 0.930)	0.906 (0.887, 0.926)	< 0.001	0.010	0.003
Bladder	0.892 (0.861, 0.923)	0.804 (0.750, 0.859)	0.903 (0.877, 0.928)	0.889 (0.856, 0.923)	0.999	0.827	0.932

Supplementary Table 3. Inference times (in second) in segmenting eight OARs by our proposed RTP-Net, U-Net, nnU-Net, and Swin UNETR. Time is represented with mean and 95% CI. Statistical analyses are performed using two-way ANOVA followed by Dunnett's multiple comparison tests. The number of eight organs can be referred to Supplementary Fig. 1. All two-tailed adjusted p values between RTP-Net and other three methods in eight organs are lower than 0.001.

	RTP-Net	U-Net	nnU-Net	Swin UNETR	$P_{(RTP\text{-}Net\ vs.\ U\text{-}Net)}$	$P_{(RTP\text{-}Net\ vs.\ nnU\text{-}Net)}$	$P_{(RTP\text{-}Net\ vs.\ Swin\ UNETR)}$
Brain	0.48<br>(0.44, 0.52)	86.27<br>(69.97, 102.57)	328.30<br>(224.54, 432.06)	70.84<br>(50.33, 91.36)	< 0.001	< 0.001	< 0.001
Brainstem	0.13<br>(0.11, 0.14)	81.58<br>(71.03, 92.13)	256.93<br>(196.06, 317.80)	62.60<br>(50.41, 74.79)	< 0.001	< 0.001	< 0.001
Rib	4.87<br>(4.65, 5.09)	48.10<br>(46.69, 49.52)	1033.82<br>(947.71, 1119.93)	19.24<br>(17.77, 20.71)	< 0.001	< 0.001	< 0.001
Heart	0.51<br>(0.48, 0.53)	68.22<br>(56.66, 79.78)	1573.83<br>(1036.13, 2111.54)	38.28<br>(28.60, 47.96)	< 0.001	< 0.001	< 0.001
Liver	1.08<br>(1.03, 1.13)	46.70<br>(45.23, 48.17)	761.61<br>(699.95, 823.28)	20.79<br>(19.29, 22.30)	< 0.001	< 0.001	< 0.001
Pelvis	1.28<br>(1.16, 1.39)	119.24<br>(105.85, 132.63)	1845.30<br>(1486.18, 2204.41)	57.88<br>(48.71, 67.06)	< 0.001	< 0.001	< 0.001
Rectum	0.32<br>(0.31, 0.33)	164.76<br>(155.90, 173.62)	1163.37<br>(1102.64, 1224.10)	159.98<br>(151.43, 168.52)	< 0.001	< 0.001	< 0.001
Bladder	0.23<br>(0.21, 0.25)	85.31<br>(74.36, 96.26)	1379.01<br>(1083.79, 1674.23)	161.54<br>(135.26, 187.82)	< 0.001	< 0.001	< 0.001

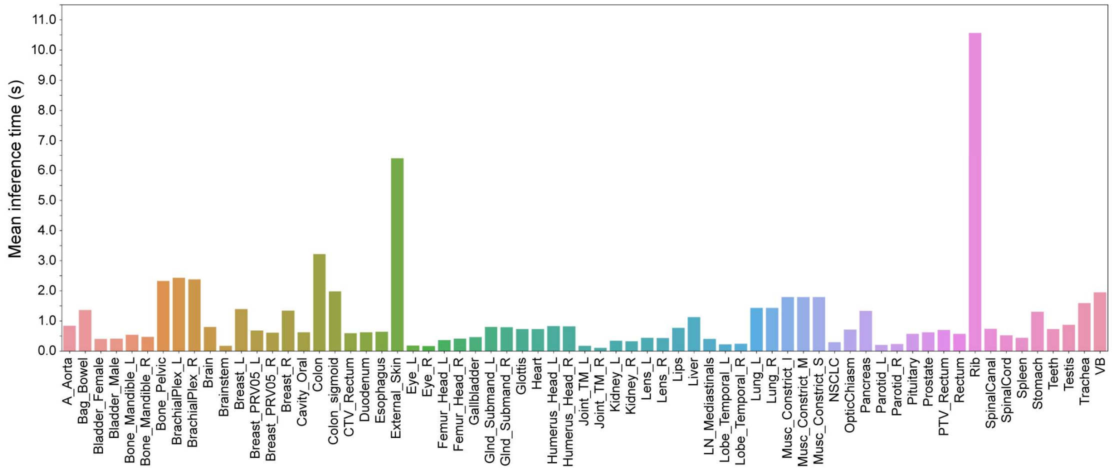

Supplementary Fig. 2. Mean inference time of RTP-Net on 67 delineation tasks.

Supplementary Table 4. Dice coefficients and inference times (in second) of four methods in target volume delineation. All descriptions are represented with mean and 95% CI. Statistical analyses are performed using two-way ANOVA followed by Dunnett's multiple comparison tests, with n = 10 replicates per condition. The two-tailed adjusted *p* values of Dice coefficients between RTP-Net and other three methods (U-Net, nnU-Net, and Swin UNETR) are 0.420, 0.999, and 0.166 for CTV segmentation, respectively, while 0.951, 0.859, and 0.832 for PTV segmentation, respectively. All two-tailed adjusted p values of inference times between RTP-Net and other three methods are lower than 0.001.

		RTP-Net	U-Net	nnU-Net	Swin UNETR	$P_{(RTP\text{-}Net\ vs.\ U\text{-}Net)}$	$P_{(RTP\text{-}Net\ vs.\ nnU\text{-}Net)}$	$P_{(RTP\text{-}Net\ vs.\ Swin\ UNETR)}$
Dice coefficient	CTV	0.910 (0.897, 0.923)	0.893 (0.866, 0.919)	0.911 (0.902, 0.920)	0.885 (0.857, 0.913)	0.420	0.999	0.166
	PTV	0.916 (0.908, 0.924)	0.910 (0.882, 0.939)	0.925 (0.918, 0.932)	0.907 (0.874, 0.939)	0.951	0.859	0.832
Inference time (s)	CTV	0.40 (0.36, 0.44)	108.41 (93.80, 123.02)	248.43 (195.36, 301.50)	62.63 (53.21, 72.05)	< 0.001	< 0.001	< 0.001
	PTV	0.44 (0.40, 0.48)	109.89 (95.10, 124.68)	119.01 (93.33, 144.70)	92.65 (80.56, 104.74)	< 0.001	< 0.001	< 0.001

Supplementary Table 5. Imaging datasets used in this study.

Dataset	Acquisition site	Region	Scanner type	Organ part
TCIA[1]				
• Head-Neck-PET-CT	Hôpital général juif (HGJ) de Montréal, QC, Canada; Centre hospitalier universitaire de Sherbrooke (CHUS), QC, Canada; Hôpital Maisonneuve-Rosemont (HMR) de Montréal, QC, Canada; Centre hospitalier de l'Université de Montréal (CHUM), QC, Canada	North America	GE	Head
• NSCLC Radiogenomics[2]	Stanford University Medical Center; Palo Alto Veterans Affairs Healthcare System	North America	GE	Chest
• A whole-body CT dataset	University Hospital Tübingen, Germany	Europe	Siemens Biograph mCT	ALL
HaN (MICCAI 2015)[3,4]	Harvard Medical School, Massachusetts General Hospital, USA	North America	Anonymous	Head
SegTHOR 2019[5]	Henri Becquerel Center (CHB), Rouen, France	Europe	Anonymous	Chest
CHAOS 2019[6]	Dokuz Eylul University Hospital, Izmir, Turkey	Europe	Philips SecuraCT; Philips Mx8000 CT; Toshiba; AquilionOne	Chest; Abdomen

MSD (MICCAI 2018)[7]	Ludwig Maximilian University of Munich, Germany;<br>Radboud University Medical Center of Nijmegen, The Netherlands;<br>Polytechnique and CHUM Research Center Montreal, Canada;<br>Tel Aviv University, Israel;<br>Sheba Medical Center, Israel;<br>IRCAD Institute Strasbourg, France;<br>Hebrew University of Jerusalem, Israel;<br>Memorial Sloan Kettering Cancer Center, USA	Europe;<br>North America;<br>Asia	GE	Chest;<br>Abdomen
LUNA16[8]	Weill Cornell Medical College, USA;<br>University of California, Los Angeles, USA;<br>University of Chicago, USA;<br>University of Lowa, USA;<br>University of Michigan, USA	North America	GE;<br>Philips;<br>Siemens;<br>Toshiba	Chest (NSCLC)
Local dataset	Fudan University Shanghai Cancer Center	Asia	uRT-linac 506c	Tumor volume (CTV and PTV)

Supplementary Table 6. Patient demographics of the imaging datasets.

Dataset	Patient age (years)	Patient sex
TCIA[1]		
• Head-Neck-PET-CT	63 (18 ~ 90)	Male 76%; Female 24%
• NSCLC Radiogenomics[2]	68 (24 ~ 87)	Male 64%; Female 36%
• A whole-body CT dataset	59 (11 ~ 95)	Male 44%; Female 56%
HaN (MICCAI 2015)[3,4]	57 (31 ~ 79)	Male 88%; Female 12%
SegTHOR 2019[5]	Anonymous	Anonymous
CHAOS 2019[6]	45 (18 ~ 63)	Male 55%; Female 45%
MSD[7]	Anonymous	Anonymous
LUNA16[8]	59 (14 ~ 85)	Male 51%; Female 49%
Local dataset	Anonymous	Anonymous

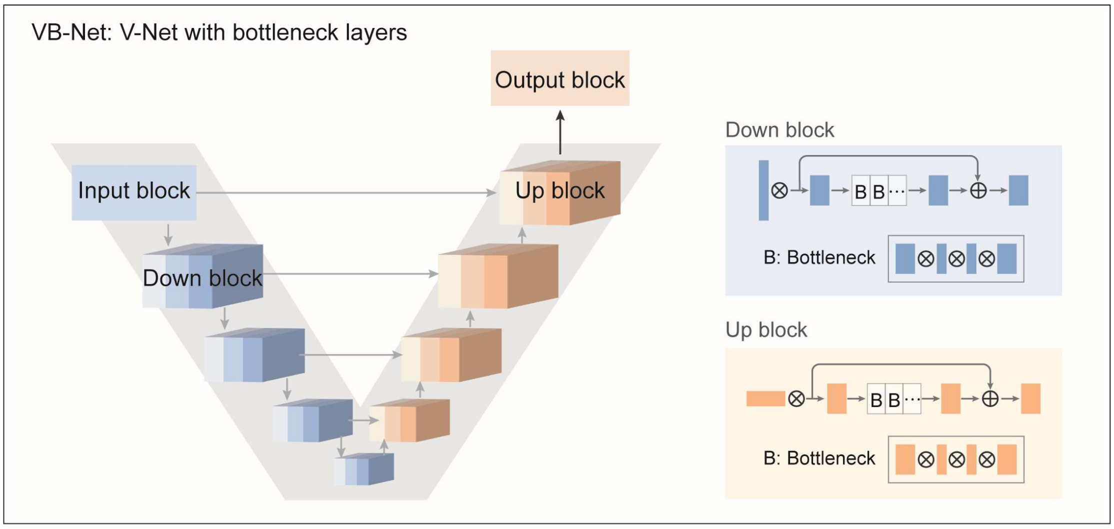

Supplementary Fig. 3. The architecture of VB-Net.

Supplementary References

1 Clark, K. et al. The Cancer Imaging Archive (TCIA): Maintaining and operating a public information repository. *J. Digit. Imaging* **26**, 1045-1057 (2013).

2 Bakr, S. et al. Data descriptor: A radiogenomic dataset of non-small cell lung cancer. *Sci. Data* **5**, 180202 (2018).

3 Raudaschl, P. F. et al. Evaluation of segmentation methods on head and neck CT: Auto-Segmentation Challenge 2015. *Med. Phys.* **44**, 2020-2036 (2017).

4 Ang, K. K. et al. Randomized phase III trial of concurrent accelerated radiation plus cisplatin with or without cetuximab for stage III to IV head and neck carcinoma: RTOG 0522. *J. Clin. Oncol.* **32**, 2940-2950 (2014).

5 Lambert, Z., Petitjean, C., Dubray, B. & Ruan, S. SegTHOR: Segmentation of Thoracic Organs at Risk in CT images. *arXiv* preprint arXiv: 1912.05950 (2019).

6 Kavur, A. E. et al. CHAOS Challenge - Combined (CT-MR) Healthy Abdominal Organ Segmentation. *Med. Image Anal.* **69**, 101950 (2021).

7 Simpson, A. L. et al. A large annotated medical image dataset for the development and evaluation of segmentation algorithms. *arXiv* preprint arXiv: 1902.09063 (2019)

8 Armato S. G. et al. The Lung Image Database Consortium (LIDC) and Image Database Resource Initiative (IDRI): A completed reference database of lung nodules on CT scans. *Med. Phys.* **38**, 915-931 (2011).s
