# Supplementary material for: Deep learning empowered volume delineation of whole-body organs-at-risk for accelerated radiotherapy
Source: Nat Commun. 2022 Nov 2;13:6566. doi: 10.1038/s41467-022-34257-x (PMC9630370; doi:10.1038/s41467-022-34257-x)
Supplement: Supplementary file 2 — Reporting Summary [file 41467_2022_34257_MOESM2_ESM.pdf]

nature portfolio

Corresponding author(s): Dinggang Shen, Yaozong Gao

Last updated by author(s): Oct 19, 2022

Reporting Summary

Nature Portfolio wishes to improve the reproducibility of the work that we publish. This form provides structure for consistency and transparency in reporting. For further information on Nature Portfolio policies, see our Editorial Policies and the Editorial Policy Checklist.

Statistics

For all statistical analyses, confirm that the following items are present in the figure legend, table legend, main text, or Methods section.

n/a	Confirmed	
☐	☒	The exact sample size (*n*) for each experimental group/condition, given as a discrete number and unit of measurement
☐	☒	A statement on whether measurements were taken from distinct samples or whether the same sample was measured repeatedly
☐	☒	The statistical test(s) used AND whether they are one- or two-sided<br>*Only common tests should be described solely by name; describe more complex techniques in the Methods section.*
☐	☒	A description of all covariates tested
☐	☒	A description of any assumptions or corrections, such as tests of normality and adjustment for multiple comparisons
☐	☒	A full description of the statistical parameters including central tendency (e.g. means) or other basic estimates (e.g. regression coefficient) AND variation (e.g. standard deviation) or associated estimates of uncertainty (e.g. confidence intervals)
☐	☒	For null hypothesis testing, the test statistic (e.g. *F*, *t*, *r*) with confidence intervals, effect sizes, degrees of freedom and *P* value noted<br>*Give P values as exact values whenever suitable.*
☒	☐	For Bayesian analysis, information on the choice of priors and Markov chain Monte Carlo settings
☐	☒	For hierarchical and complex designs, identification of the appropriate level for tests and full reporting of outcomes
☒	☐	Estimates of effect sizes (e.g. Cohen's *d*, Pearson's *r*), indicating how they were calculated

Our web collection on statistics for biologists contains articles on many of the points above.

Software and code

Policy information about availability of computer code

Data collection	We used 3D Slicer 5.0.2 for data annotation, ITK-SNAP 3.8.0 for data visualization, and the platform of PyTorch with one Nvidia Tesla V100 GPU for deep learning system design. The related code is available at GitHub (https://github.com/simonsf/RTP-Net).
Data analysis	We used IBM SPSS 26.0 for statistical analyses, GraphPad Prism 9 and Adobe Illustrator CC 2019 for figure plot.

For manuscripts utilizing custom algorithms or software that are central to the research but not yet described in published literature, software must be made available to editors and reviewers. We strongly encourage code deposition in a community repository (e.g. GitHub). See the Nature Portfolio guidelines for submitting code & software for further information.

Data

Policy information about availability of data

All manuscripts must include a data availability statement. This statement should provide the following information, where applicable:

- Accession codes, unique identifiers, or web links for publicly available datasets
- A description of any restrictions on data availability
- For clinical datasets or third party data, please ensure that the statement adheres to our policy

The OAR-related images (N = 28,219) that support experiments in this paper came from the publicly available multi-center datasets, i.e., The Cancer Imaging Archive (TCIA, https://www.cancerimagingarchive.net/), Head and Neck (HaN) Autosegmetation Challenge 2015 (https://paperswithcode.com/dataset/miccai-2015-head-and-neck-challenge), Segmentation of Thoracic Organs at Risk in CT Images (SegTHOR) Challenge 2019 (https://segthor.grand-challenge.org/), Combined (CT-MR) Healthy Abdominal Organ Segmentation (CHAOS) Challenge 2019 (https://chaos.grand-challenge.org/), Medical Segmentation Decathlon (MSD) Challenge 2018

(http://medicaldecathlon.com/), and LUng Nodule Analysis (LUNA) 2016 (https://luna16.grand-challenge.org/). The rest tumor-related data (N = 362) were obtained from Fudan University Shanghai Cancer Center (Shanghai, China), where partial data (i.e., 50 cases) are released together with the code, with the permission obtained from respective cancer center. The full dataset are protected because of privacy issues and regulation policies in cancer center.

Human research participants

Policy information about studies involving human research participants and Sex and Gender in Research.

Reporting on sex and gender	Sex and gender were equally considered in the study design. This deep learning-based image segmentation could be suitable for people of all sexes and genders.
Population characteristics	The details of age and gender were provided in Supplementary Table 6.
Recruitment	This was a retrospective study with no specific recruitment requirements.
Ethics oversight	This study was approved by the Research Ethics Committee in Fudan University Shanghai Cancer Center, Shanghai, China (No. 2201250-16).

Note that full information on the approval of the study protocol must also be provided in the manuscript.

Field-specific reporting

Please select the one below that is the best fit for your research. If you are not sure, read the appropriate sections before making your selection.

☒ Life sciences ☐ Behavioural & social sciences ☐ Ecological, evolutionary & environmental sciences

For a reference copy of the document with all sections, see nature.com/documents/nr-reporting-summary-flat.pdf

Life sciences study design

All studies must disclose on these points even when the disclosure is negative.

Sample size	We adopted a large-scale dataset of 28,581 CT scans, of which ~83% was used as the training set for 67 segmentation tasks. Therefore, the average number of cases for each segmentation task in the training phase was 354. We followed the state-of-the art articles, where 132 cases in average were used for each of 23 segmentation tasks (Isensee, F. et al. 2021).
Data exclusions	No data was excluded.
Replication	The deep learning model (RTP-Net) was repeated five times, and the performances were average as the final results.
Randomization	We randomly splited our dataset as training and testing sets in Figure S1, where testing set accounted for ~17%.
Blinding	The process was blinding.

Reporting for specific materials, systems and methods

We require information from authors about some types of materials, experimental systems and methods used in many studies. Here, indicate whether each material, system or method listed is relevant to your study. If you are not sure if a list item applies to your research, read the appropriate section before selecting a response.

Materials & experimental systems

n/a	Involved in the study	
☒	☐	Antibodies
☒	☐	Eukaryotic cell lines
☒	☐	Palaeontology and archaeology
☒	☐	Animals and other organisms
☒	☐	Clinical data
☒	☐	Dual use research of concern

Methods

n/a	Involved in the study	
☒	☐	ChIP-seq
☒	☐	Flow cytometry
☒	☐	MRI-based neuroimaging
